# Correlations between Coffee Consumption and Metabolic Phenotypes, Plasma Folate, and Vitamin B12: NHANES 2003 to 2006

**DOI:** 10.3390/nu13041348

**Published:** 2021-04-18

**Authors:** Pratibha V. Nerurkar, Krupa Gandhi, John J. Chen

**Affiliations:** 1 Laboratory of Metabolic Disorders and Alternative Medicine, Department of Molecular Biosciences and Bioengineering (MBBE), College of Tropical Agriculture and Human Resources (CTAHR), University of Hawaii at Manoa, Honolulu, HI 96822, USA; 2 Division of Biostatistics, Thomas Jefferson University Hospitals, Philadelphia, PA 19107, USA; krupa.gandhi@jefferson.edu; 3 Department of Quantitative Health Sciences, John A. Burns School of Medicine, University of Hawaii at Manoa, Honolulu, HI 96813, USA; jjchen@hawaii.edu

**Keywords:** coffee, metabolic healthy obesity, metabolically healthy phenotype, metabolically unhealthy phenotypes

## Abstract

Metabolic syndrome (MetS) is prevalent not only among the overweight and obese but also normal weight individuals, and the phenotype is referred to as a metabolically unhealthy phenotype (MUHP). Besides normal weight individuals, overweight/obese individuals are also protected from MetS, and the phenotype is known as a metabolically healthy phenotype (MHP). Epidemiological studies indicate that coffee and micronutrients such as plasma folate or vitamin B12 (vit. B12) are inversely associated with MetS. However, correlations among coffee consumption metabolic phenotypes, plasma folate, and vit. B12 remain unknown. Our objective was to investigate the correlation between coffee consumption, metabolic phenotypes, plasma folate, and vit. B12 as well as to understand associations between plasma folate, vit. B12, and metabolic phenotypes. Associations among coffee consumption metabolic phenotypes, plasma folate, and vit. B12 were assessed in a cross-sectional study of 2201 participants, 18 years or older, from 2003–2004 and 2005–2006 National Health and Nutrition Examination Surveys (NHANES). MUHP was classified as having > three metabolic abnormalities. Coffee consumption was not associated with metabolic phenotypes, but negatively correlated with several metabolic variables, including BMI (*p* < 0.001). Plasma folate was positively associated with MUHP (*p* < 0.004), while vit. B12 was inversely associated with MUHP (*p* < 0.035). Our results suggest the potential protective impact of coffee on individual components of MetS and indicate a positive correlation between coffee consumption and MUHP among overweight individuals. Identifying possible dietary factors may provide practical and low-cost dietary intervention targets, specifically for early intervention. Larger and randomized intervention studies and prospective longitudinal studies are required to further evaluate these associations.

## 1. Introduction

Obesity is prevalent in about 36.5% of the U.S. adult population (ages 20–74 years) [1] and associated with a high risk of metabolic syndrome (MetS), cardiovascular diseases (CVD), type 2 diabetes (T2D), certain cancers, and other causes of preventable deaths [2]. MetS, as defined by International Diabetes Federation and Karelis et al., includes an index of insulin resistance such as homeostatic model assessment of insulin resistance (HOMA-IR), hyperlipidemia risk factors such as low levels of high-density lipoproteins (HDL), high plasma levels of triglycerides or low-density lipoprotein (LDL), markers of inflammation such as high-sensitivity C-reactive protein (hsCRP), increased levels of plasma homocysteine (Hcy), abnormal plasma glucose, abnormal blood pressure as well as high levels of visceral and/or liver fat [3,4]. In the past decade a sub-set of the obese population have been identified who are “protected” from MetS, and the phenotype is classified as “metabolically healthy obesity (MHO)” [5]. Initially defined by Sims in 2001 [6], MHO is also referred to as metabolically healthy phenotype (MHP) and is identified by an absence of MetS [3,4,7,8]. There is no general consensus regarding the definition of MHP. Therefore, based on the definition of obesity, metabolic markers, and their cut-off point criteria, the prevalence of MHP varies significantly among the adult U.S. population and ranges from 3.3 to 57.5% [3,9]. A recent study by Al-Khalidi et al. and Winter et al. defined MHP as obese individuals with zero metabolic abnormality [9,10]. Some studies have also included deficiencies of plasma vitamin D and ferritin levels as part of MetS classification, and both plasma vitamin D and ferritin are found be inversely associated with MHP [9,11,12]. It has been suggested that MHP may be transient, with genetics and lifestyle factors substantially contributing towards transitioning from MHP to MUHP. However, protective factors that promote MHP are not well studied [13]. One recent prospective study indicated that among several dietary factors, higher coffee intake was associated with a reduced risk of developing MUHP among normal weight, overweight, and obese individuals [14]. 

For more than two decades, coffee consumption has risen worldwide and is the most consumed beverage in the United States, even more than water [15,16]. Habitual coffee consumption is found to be inversely associated with total and cause-specific mortality as well as several chronic diseases [17,18,19,20,21,22,23]. Besides caffeine, brewed coffee contains several bioactive compounds including polyphenols and diterpene alcohols such as cafestol and kahweol, which are associated with lower incidences of metabolic syndrome and CVD risk factors including high blood pressure, plasma cholesterol, and homocysteine (Hcy) [15,17,24,25]. Epidemiological studies indicate that higher coffee consumption reduced the genetic associations with body mass index (BMI) and obesity risk and type 2 diabetes [26,27,28]. Gene–diet interaction analysis in three US prospective studies indicated that the higher consumption of coffee was associated with reduced obesity risk among individuals who were genetically predisposed to obesity as measured by 77 BMI-associated single nucleotide polymorphisms (SNPs) [26]. Similarly, in a weight loss intervention study, the genetic risk factors consisting of eight habitual coffee consumption-associated SNPs related to coffee and glucose metabolism was reduced by habitual coffee consumption [27]. In contrast, a pooled analysis of four Korean prospective studies with 71,527 participants indicated that consumption of more than three cups of coffee/day significantly reduced the risk of T2D but was not associated with five SNPs associated with T2D [28]. Several studies identified a positive association between coffee intake and reduced incidence of MetS, while other studies did not [29,30,31,32,33]. Inconsistencies in the correlations between coffee consumption and MetS could stem from type (caffeinated vs. decaffeinated) and variety of coffee, amount of coffee (cups/day), other ingredients such as milk, cream, and sugar added to coffee or confounding factors such as smoking [18,34]. Cytochrome (CYP) P450 1A2 is the enzyme involved in breakdown or metabolizing caffeine. Studies indicate that interindividual variations in CYP P450 1A2 may partly be responsible for the inconsistent associations between habitual coffee consumption and disease risks [35,36,37].

Mechanisms by which coffee may reduce MetS are not clearly understood but may be associated in part with the ability of chlorogenic acids (CGAs) and other bioactive components in coffee, rather than caffeine, to reduce intestinal glucose absorption or improve insulin sensitivity or the association of coffee consumption with improved levels of leptin and ghrelin, which regulate appetite, satiety, and energy metabolism [38,39,40,41,42,43,44,45,46,47]. 

Elevated plasma levels of hyperhomocysteinemia (Hcy) have been suggested to be both a cause and a consequence of the MetS and is associated with smoking habits, alcohol and caffeine abuse, or a diet low in fruits and vegetables. However, association of hyperhomocysteinemia with overall coffee intake is inconsistent. Study by Miranda et al. indicated that coffee intake of 1–3 cups/day and its polyphenols were inversely associated with hyperhomocysteinemia [24], while other studies, including those by Grubben et al., demonstrated that consumption of more than two cups of coffee/day was associated with increased levels of plasma Hcy [48,49,50]. Hcy is a sulfur-containing amino acid whose plasma levels in humans are correlated with not only deficiencies, but also suboptimal nutritional status of vitamin B6, B12, and folate, which serve as cofactors and methyl donors in Hcy metabolism [50,51]. Coffee and chlorogenic acid consumption significantly increased plasma Hcy levels, while independent studies indicate negative correlations between coffee consumption and plasma folate and vit. B12 [52,53,54,55,56,57]. 

The primary goal of our study was to determine the association between coffee consumption and MHP (defined by <3 cardiometabolic abnormalities) and MUHP (defined by >3 cardiometabolic abnormalities) using data from the Continuous National Health and Nutritional Examination Survey (NHANES; 2003–2004 and 2005–2006). Our secondary objective was to understand the correlation between coffee intake and modulators of Hcy metabolism, specifically plasma folate and vit. B12, and the modulators’ possible associations with metabolic phenotypes. 

## 2. Materials and Methods

### 2.1. Study Population

NHANES is a population-based cross-sectional survey, which examines the health and nutritional status of children and adults in the United States. A random sampling method is used to select participants. The survey involves a home interview and a medical exam. The home interview comprises questions related to demographics, socioeconomic status as well as dietary and health information. Medical exams include dental and physical exams, physiological measurements, and laboratory tests. Public use datasets for the ongoing NHANES are released by the National Center for Health Statistics (NCHS) of Center for Disease Control and Prevention (CDC) in two-year cycles. The NCHS ethics committee approved NHANES study protocol. Details for the NHANES study design are published elsewhere [58]. Data for this project were obtained from Continuous NHANES Data, Questionnaires, and Related Documentation for the years 2003–2004 and 2005–2006. The time frame was chosen since NHANES stopped collecting data on vit. B12 and folate after 2006.

**Figure 1:**
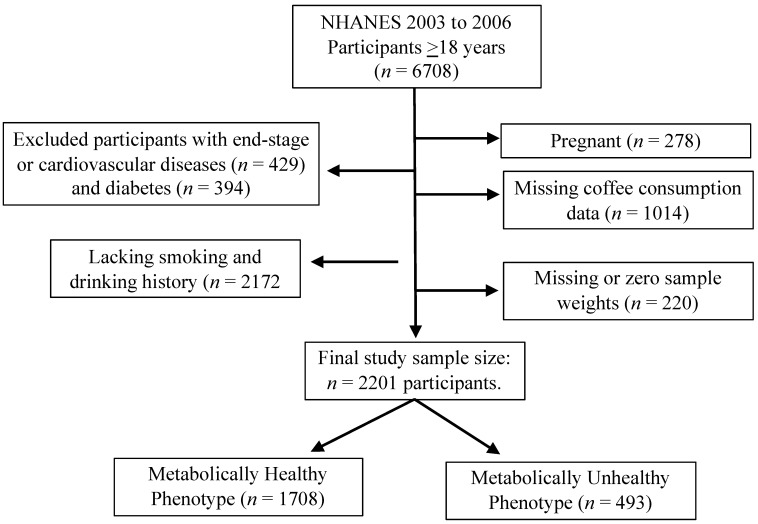
Study participant selection criteria, NHANES 2003 to 2006.

Participants 18 years and older were included in the study. NHANES 2003–2004 and 2005–2006 included a total of 6708 participants (Figure 1). Since the objective was to understand the correlation between coffee intake and metabolic phenotypes (MHP and MUHP) and not the end stage diseases, participants were excluded based on their cardiovascular diseases status such as congestive heart failure, coronary heart disease, angina, heat attack or stroke (*n* = 429), and diabetes (*n* = 394). In addition, those who lacked smoking and drinking history (*n* = 2172), were pregnant (*n* = 278), missing coffee consumption data (*n* = 1014), and missing or zero sample weights (*n* = 220) were also excluded from the analysis. Final study sample size was *n* = 2201 participants (1054 males and 1147 females). 

### 2.2. Data Collection

Twelve-month food frequency questionnaires and 24-h dietary recalls collected from NHANES 2003–2004 and 2005–2006 were used to obtain caffeine and coffee consumption data [58]. Caffeine consumption included all sources of caffeine drinks, such as tea, soft drinks, energy drinks, etc. Participants consuming caffeinated coffee were included, while participants consuming decaffeinated coffee were not considered in our study. All types of coffee brewing methods were included in the dataset such as coffee brewed at home, cafes or restaurants, black coffee or those with cream or sugar, filter coffee, expresso, cold brew, etc. The frequency of coffee consumption was reported as <1 cup/month, 1–3 cups/month, 1 cup/week, 2–4 cups/week, 5–6 cups/week, 1 cup/day, 2–3 cups/day, 4–5 cups/day, or ≥6 cups/day, based on the 24-h dietary recall. It was further categorized as none or ≤1 cup/week, 2–6 cups/week, 1 cup/day, and ≥2 cups/day (1 cup = 8 oz) for analysis. 

### 2.3. Classification of MetS, Metabolically Healthy, and Unhealthy Phenotypes

Based on American Heart Association/National Heart, Lung and Blood Institute (AHA/NHLB) criteria for MetS diagnosis, an individual should have a minimum of three out of five metabolic abnormalities that includes: (1) waist circumference ≥90 cm for men and ≥80 cm for women; (2) triglyceride ≥ 150 mg/dL or medication for hyperlipidemia; (3) HDL cholesterol < 40 mg/dL for men and <50 mg/dL for women; (4) blood pressure ≥ 130/85 mmHg or medication for hypertension; (5) fasting blood glucose ≥ 100 mg/dL or taking glucose lowering medication [59]. There is currently no consensus about the definition of MHP and MUHP, and it varies based on the criteria as well as cutoff values, thereby influencing the prevalence and outcomes [9,10,60]. Overall, MHP is defined as obese individuals with normal fasting glucose, insulin sensitivity, blood lipid profiles, blood pressure, and absence of MetS [61,62]. 

MHP in our study was based on the definition of MetS and defined as those individuals with less than three metabolic abnormalities, and MUHP was classified as those participants with more than or equal to three metabolic abnormalities, independent of their BMI status. We also evaluated the effects of smoking, physical activity, and alcohol on metabolic phenotypes, since these health behaviors are known risk factors for metabolic syndrome [63,64]. 

### 2.4. Plasma Folate and Vitamin B12

The NHANES questionnaire and phlebotomy exam files are linked to a laboratory data file by using the unique survey participant identifier SEQN. The Phlebotomy Examination file contains information on duration of fasting, time of blood draw, and the conditions that would exclude the blood draw. Plasma or serum are analyzed at the Division of Environmental Health Laboratory Sciences, National Center for Environmental Health, and Centers for Disease Control and Prevention (https://wwwn.cdc.gov/Nchs/Nhanes/2005-2006/B12_D.htm (accessed on 20 September 2020)). 

Both serum folate and vitamin B12 were measured by using the Bio-Rad Laboratories “Quantaphase II Folate/Vitamin B12” radio-assay kit. Specimen collection and processing instructions are specified on the NHANES website (https://wwwn.cdc.gov/Nchs/Nhanes/2005-2006/B12_D.htm (accessed on 15 September 2020)). Plasma folate quartiles were data driven and were categorized as <8.3, 8.3–<11.5, 11.5–<16.1, ≥16.1 ng/mL. Plasma vit. B12 was categorized based on quartiles <359.0, 359.0–<470.5, 470.5–<634.5, and ≥634.5 pg/mL, as published [65]. 

### 2.5. Covariates 

Additional covariates included in the analysis were demographic variables such as age group (categorized as 20–34, 35–49, 50–64, 65–79, and ≥80 years), sex (male vs. female), race/ethnicity (categorized as non-Hispanic White, Mexican American or Hispanic, Non-Hispanic Black, and other). Health behavior variables included smoking status (categorized as never, former, or current smoker), alcohol drinker (categorized as never, former or current drinker, where current drinker was further categorized as <1 drink/day, 1–2 drinks/day, and >2 drinks/day), and physical activity (metabolic equivalent of task, MET, categorized as <5, 5–19.9, 20–49.9, and ≥50 h/week). MET is a unit to estimate energy expenditure above resting metabolic rate during all levels of physical activity (light, moderate, heavy) including walking [66]. Other metabolic characteristics include body mass index (BMI, categorized as normal, overweight, and obese), triglycerides, low-density lipoprotein (LDL-c), high-density lipoproteins (HDL-c), C-reactive protein (CRP), and homeostasis model assessment-estimated insulin resistance (HOMA-IR). BMI was calculated from the physical examination survey that recorded anthropometric data for height, weight, and waist circumference. Smoking history, alcohol consumption and physical activity data were collected from the lifestyle questionnaire. Details for each variable measurement is published on the NHANES website [67].

### 2.6. Statistical Analysis

The survey data were summarized by descriptive statistics, followed by one-way analysis of variance (ANOVA) for continuous variables and Rao–Scott chi-square tests for categorical variables to assess associations between metabolic phenotypes and other factors. The analysis accounted for the stratified, multistage probability cluster sampling design of NHANES. NHANES provides sampling weights to be used in analyses that account for oversampling of certain subgroups [67]. Coffee consumption was the primary independent variable, and metabolic phenotypes were the main outcome variable. We also assessed the relationships between B12 and folate with the outcome, and adjusted them in the main analysis (between coffee and metabolic phenotypes) and stratified by BMI status. Associations between micronutrients and coffee consumption were also assessed. Variables with *p* < 0.1 in the bivariate analysis were included in the multivariable logistic regression analysis and backward selection method was performed to determine final significant variables keeping the primary independent variables of interest in the model. Logistic analyses were performed to model the association between metabolic phenotypes (outcome) and coffee intake. Odds ratios (ORs) and their 95% confidence intervals (CIs) were calculated. All analyses accounted for NHANES’ complex multistage sampling design, and *p* < 0.05 was considered statistically significant. Statistical analyses were conducted using SAS software, version 9.4 (SAS Institute Inc., Cary, NC, USA).

## 3. Results

### 3.1. Baseline Characteristics of Participants

General characteristics of coffee drinkers for NHANES 2003–2006 has been described by Loftfield et al. [58]. In our study, among the 2201 participants analyzed in the 2003–2006 NHANES data, 1708 (77.6%) of the individuals demonstrated MHP, while 493 (22.4%) individuals demonstrated an unhealthy phenotype (Table 1). Younger age, sex (female), and Mexican American and non-Hispanic Black were significantly associated with a healthy phenotype (Table 1, all *p*-values < 0.001). As expected, smoking status and number of alcoholic drinks consumed were negatively associated with MHP, while physical activity was positively associated with MHP (Table 1, *p* = 0.002, *p* < 0.001 and *p* = 0.003, respectively). All five MetS components—waist circumference, blood pressure, glucose, HDL-c, and triglyceride—were negatively correlated with MHP (Table 1, all *p*-values < 0.001). Similarly, HOMA-IR components, BMI, and total cholesterol were negatively correlated with MHP (Table 1, all *p*-values < 0.001). Our analysis indicated that LDL-c and CRP were not associated with metabolic phenotypes (Table 1). 

### 3.2. Correlations between Coffee Intake Frequencies and Metabolic Phenotypes and Individual Metabolic Variables

Among our overall study sample, a total of 1292 (58.7%) participants were coffee drinkers (Table 2). It was noted that, among the coffee drinkers, 683 (71.8%) individuals drank ≥ two cups of coffee/day (Table 2). We did not find any significant correlations between the level of coffee consumption and metabolic phenotypes (Table 2, *p* = 0.513). Similarly, no significant difference in overall caffeine intake was observed between the metabolic phenotypes (Table 2, *p* = 0.267). 

As noted in Table 3, several independent metabolic variables were significantly associated with increased coffee consumption. Consumption of more coffee per day was significantly associated with reduced BMI (Table 3, *p* < 0.001). Additionally, increased coffee intake (one to two cups of coffee/day) was significantly associated with increases in systolic blood pressure (SBP, *p* = 0.027), glucose (*p* = 0.02), LDL-c (*p* < 0.001), and total cholesterol (*p* < 0.001, Table 3). Diastolic blood pressure (DBP), HDL-c, and triglycerides were unaffected by coffee consumption (Table 3, *p* = 0.568, *p* = 0.166 and *p* = 0.522, respectively). HOMA-IR and CRP were significantly reduced with increased coffee consumption (Table 3, *p* < 0.001 and *p* = 0.021, respectively). However, no significant correlation was noted between the amount of coffee consumed and the number of metabolic abnormalities observed (Table 3, *p* = 0.871).

It was interesting to note that, when the study population was stratified by BMI status (Table 4), increased coffee consumption was significantly associated with metabolic phenotypes among the overweight individuals (Table 3, *p* = 0.025). After stratifying for BMI status, overall caffeine intake still was not different between metabolic phenotypes (Table 3, normal: *p* = 0.27; overweight: *p* = 0.96, obese: *p* = 0.38).

### 3.3. Correlations between Micronutrients and Coffee Intake and Metabolic Phenotypes

In the current sample, increased coffee consumption from none/≤1 cup/week to ≥2 cups/day was found to be significantly associated with increased plasma folate levels (Table 4, *p* < 0.009), while not significantly associated with plasma vit. B12, either as a continuous or a categorical variable (Table 3, *p* = 0.218 and *p* = 0.475, respectively). Table 5 indicates that the overall total plasma folate was positively associated with MUHP but not as a categorical variable (*p* = 0.004 and *p* = 0.277, respectively). Similarly, lower plasma level of vit. B12 was significantly associated with an MUHP, both as a continuous and a categorical variables (*p* = 0.035 and *p* = 0.027, respectively). It was interesting to note that, based on BMI status, increased plasma folate was significantly correlated to MUHP among the overweight and obese individuals (Table 6, *p* = 0.001 and *p* = 0.003, respectively). Plasma vit. B12 levels were not correlated with metabolic phenotypes based on BMI status (Table 6, with *p*-values ranging from 0.44 to 0.57).

## 4. Discussion

### 4.1. Correlation between Coffee Intake Frequencies and Metabolic Phenotypes

While BMI is the most widely used obesity index, it does not necessarily reflect the risk of developing metabolic disorders [68,69,70]. An increasing number of studies have identified the existence of MHP among overweight and obese individuals as well as MUHP among normal weight or underweight individuals [71]. There is no universal definition to categorize these phenotypes and could include a varying number of cardiometabolic risk factors [10]. Based on the definition of MetS that includes ≥3 cardiometabolic risk factors, about 22% of our study population demonstrated MUHP, while based on BMI (≥30 Kg/m^2^) about 66% demonstrated an MUHP. A study by Winter et al. using the same NHANES survey data (2003–2004 and 2005–2006) indicated that about 49.8% of the study population demonstrated MUHP [10]. The differences in the MUHP outcomes are possibly due to the differences in the classification of MUHP (0 vs. 3 abnormalities) and/or inclusion of youth (ages 12 to 18 years) [10]. However, similar to the study by Winter et al., our study also demonstrates a positive and significant influence of physical activity on MHP [10]. 

Moderate coffee consumption (2–4 cups/day) has been indicated as a protective factor for the development of cardiometabolic risk factors [32]. Our results from NHANES survey data did not indicate any significant correlation between coffee consumption and overall metabolic phenotypes. However, when the study population was stratified based on BMI, increased coffee consumption was significantly associated with MUHP among overweight individuals. Independent studies have indicated that gene–diet interactions may be associated with lower BMI among genetically predisposed individuals who drank 1–3 cups of coffee/day [27]. Similarly, daily consumption of coffee with bread was associated with lower visceral adipose tissue and lower prevalence of visceral obesity and MetS among Japanese population [72]. However, high coffee consumption of ≥3 cups/day was positively associated with obesity among Korean women [73]. The exact mechanisms of high coffee consumption with obesity of MUHP remain unclear. However, at a molecular level, caffeine is known to influence energy balance by increasing energy expenditures and reducing energy intake [74]. Furthermore, obese individuals consuming coffee containing high chlorogenic acid for 12 weeks demonstrated reduced accumulation of visceral adipose tissue, reduced BMI, and reduced waist circumference [75].

### 4.2. Influence of Coffee Intake on Metabolic Syndrome (MetS) and Its Components

Several studies indicated an improvement of MetS or its components including high blood pressure with consumption of 2–4 cups of coffee/day ([29,31,33] and references within). However, we observed that ≥2 cups/day of coffee consumptions was significantly associated with increased plasma levels of LDL and total cholesterol, as well as systolic blood pressure (SBP). Our results are similar to those of Kim et al., which indicated that coffee consumption may increase the risk of metabolic syndrome in the Korean population [30]. Conversely, a sex effect was noted in studies by Kim et al., wherein coffee consumption reduced MetS in Korean women [31]. It is unclear if the adverse effects of coffee on LDL and total cholesterol noted in our study are related to type of coffee or additives such as sugar and/or milk/cream. Drinking more than nine cups of coffee per day was positively associated with increased serum cholesterol among several populations-based studies, including Japanese men in Hawaii [76,77]. Studies indicate that moderate paper-filtered coffee consumption for four weeks may increase plasma cholesterol among healthy individuals [78]. Diterpene cafestol, present in unfiltered coffee, is known to increase serum cholesterol levels [79,80].

Similar to studies by Wildman et al., we noted that independent cardiometabolic abnormalities such as elevated HOMA-IR, CRP, elevated triglyceride, and waist circumference were significantly associated with MUHP [5]. We further observed that consumption of ≥2 cups/day of coffee was inversely associated with cardiometabolic abnormalities including elevated HOMA-IR and CRP but not triglycerides. In our study, it was interesting to note that regardless of the number of metabolic abnormalities, coffee consumption was not associated with any metabolic phenotypes.

### 4.3. Correlation between Micronutrients, Coffee Intake, and Metabolic Phenotypes 

Elevated plasma homocysteine is a risk factor for CVD and is influenced by folate and vitamin B deficiencies including B12 [50,81]. Several studies have linked coffee consumption to increased levels of plasma homocysteine [57,81,82,83,84,85] and inconsistent correlations with plasma folate or vit. B12 [50,55,81]. In our study population, higher coffee consumption was significantly associated with higher levels of plasma folate (≥16.1 ng/mL). Our results are in contrast with published studies, which demonstrate that either coffee intake was not associated with plasma folate [49,81] or significantly lowered plasma folate in a dose-dependent manner [55]. Similar to another published study, we did not observe any association of increased coffee consumption with plasma B12 levels [55,81]. Among the same NHANES population (2003–2004 and 2005–2006), higher plasma folate levels were associated with obesity [86]. Similarly, we noted that high plasma folate levels were associated with MUHP among the overweight and obese population. In contrast, lower plasma B12 levels were associated MUHP but not with obesity. 

The public health relevance of “metabolic phenotypes” is currently unclear, due to the lack of established definition and criteria as well as its long-term clinical implications. Studies have indicated that MUHP can develop among 50% of individuals with MHP, irrespective of their weights and BMI [87,88]. Meta-analysis of epidemiological studies indicated that intentional weight loss among obese individuals with MUHP lowered all-cause mortality but had no effect on mortality among the MHP [89]. Current treatment options for obese individuals are limited and, based on the concept “one size fits all”, may be counterproductive with “metabolically healthy obese” individuals. Potentially, scarce and expensive resources can be more effectively used if tailored towards the unhealthy metabolic profile, regardless of BMI. In view of a rising obesity epidemic, stratifying individuals based upon their “metabolic phenotypes” could help to improve therapeutic decision making towards a more individualized treatment plan. Moreover, classification of metabolic phenotypes and identifying biomarkers of phenotypic transitions is also expected to detect the metabolic perturbations at an early stage and identify normal weight individuals at risk, who would otherwise elude detection due to false presumptions of being “healthy”. A prospective study by Mirmiran et al. indicated that irrespective of the BMI status, diet can influence the transition of MHP to MUHP among adults [14]. Identifying possible protective factors such as coffee, folate, or vit. B12 is expected to provide practical and low-cost dietary intervention targets, specifically for early intervention. Our results indicate that coffee could be integrated as part of a healthy diet and may have practical implications to manage chronic diseases or risk factors for metabolic syndrome.

One of the major study limitations is that this is secondary data analysis based on a national survey, and we are, therefore, limited by what variables are available and how the information was collected. In addition, this study has limitations possibly arising from the cross-sectional nature of the data, definition of metabolic phenotypes, and including only the subpopulation with data on plasma vit. B12 and folate. Our analysis did not take into consideration the type of coffee or its varieties, coffee preparation methods, ethnicity, sex, or younger population (ages 12 to 18 years). Regardless, our study suggests the association of coffee as a possible protective factor with individual components of MetS and indicates a potentially positive correlation between coffee consumption and MHP among overweight individuals.

## 5. Conclusions

Our current study indicates that overall coffee consumption was not correlated with either MHP or MUHP, but negatively correlated with several metabolic variables. Plasma folate was positively associated with MUHP, while vit. B12 was inversely associated with MUHP among our study population. 

## Figures and Tables

**Table 1:** Participant demographics, health behavior, and metabolic characteristics by metabolic phenotypes.

Variables	Metabolic Phenotypes *		Weighted *p*-Value
	Metabolic Healthy Phenotype (MHP) (*n* = 1708)	Metabolic Unhealthy Phenotype (MUHP) (*n* = 493)	
**Demographics**			
**Age Group, years (*n*, %)**			<0.001
20–34	524 (30.9%)	36 (8.8%)	
35–49	507 (36.0%)	103 (28.6%)	
50–64	341 (21.7%)	166 (37.6%)	
65–79	213 (8.2%)	151 (21.7%)	
≥80	123 (3.1%)	37 (3.3%)	
**Sex (*n*, %)**			<0.001
Male	790 (43.1%)	264 (53.8%)	
Female	918 (56.9%)	229 (46.1%)	
**Race/Ethnicity (*n*, %)**			<0.001
Non-Hispanic White	924 (73.0%)	313 (83.0%)	
Mexican American or Hispanic	353 (10.5%)	90 (5.1%)	
Non-Hispanic Black	353 (11.0%)	73 (7.1%)	
Other	78 (5.4%)	17 (4.8%)	
**Health Behaviors**			
**Smoker (*n*, %)**			0.002
Never	917 (52.5%)	232 (47.5%)	
Former	409 (22.8%)	169 (32.4%)	
Current	382 (24.8%)	92 (20.1%)	
**Alcohol Drinker (*n*, %)**			<0.001
Never	209 (11.3%)	70 (15.6%)	
Former	252 (14.1%)	92 (21.6%)	
Current: <1 drink/day	773 (56.5%)	190 (48.8%)	
1–2 drinks/day	171 (11.8%)	28 (6.4%)	
>2 drinks/day	81 (6.3%)	28 (7.6%)	
**Physical Activity** **MET hour/week (*n*, %)**			0.003
<5.0	259 (21.4%)	79 (29.6%)	
5.0–19.9	407 (36.3%)	121 (40.5%)	
20.0–49.9	290 (26.9%)	62 (21.9%)	
≥50.0	170 (15.3%)	25 (8.0%)	
**MetS Components**			
Waist circumference, cm (Mean ± SE)	92.69 ± 0.36	111.46 ± 0.75	<0.001
Waist circumference, cm (*n*, %)			<0.001
≥102	373 (21.3%)	378 (81.3%)	
<102	1294 (78.7%)	111 (18.7%)	
SBP, mm Hg (Mean ± SE)	119.18 ± 0.61	129.13 ± 0.98	<0.001
DBP, mm Hg (Mean ± SE)	69.46 ± 0.38	73.78 ± 0.72	<0.001
Elevated blood pressure, mm Hg (*n*, %)			<0.001
≥130/85 or medication use	270 (13.0%)	337 (65.3%)	
<130/85 or no medication use	1438 (87.0%)	156 (34.7%)	
Glucose, mg/dL (Mean ± SE)	92.99 ± 0.38	103.12 ± 0.51	<0.001
Elevated glucose, mg/dL (*n*, %)			<0.001
≥100 or medication use	336 (17.2%)	356 (69.9%)	
<100 or no medication use	1372 (82.8%)	137 (30.1%)	
HDL-c, mg/dL (Mean ± SE)	57.73 ± 0.65	45.66 ± 1.20	<0.001
Decreased HDL-c Level, mg/dL (*n*, %)			<0.001
<40 for men or <50 for women or medication use	215 (12.4%)	313 (62.7%)	
≥40 for men or ≥50 for women or no medication use	1493 (87.6%)	180 (37.3%)	
Triglycerides, mg/dL (Mean ± SE)	115.83 ± 2.20	208.60 ± 7.06	<0.001
Elevated triglycerides, mg/dL (*n*, %)			<0.001
≥150	307 (18.7%)	335 (69.7%)	
<150	1381 (81.3%)	155 (30.3%)	
**HOMA-IR Components**			
Insulin, µU/mL (Mean ± SE)	7.73 ± 0.22	15.92 ± 0.91	<0.001
HOMA-IR (Mean ± SE)	11.69 ± 0.32	25.06 ± 1.29	<0.001
HOMA-IR, (*n*, %)			<0.001
Quartile 4	271 (14.2%)	245 (53.1%)	
Quartiles 1 to 3	1411 (85.8%)	243 (46.9%)	
**Other Metabolic Characteristics**			
BMI (Kg/m^2^, Mean ± SE)	26.86 ± 0.14	33.17 ± 0.38	<0.001
BMI (Kg/m^2^, *n*, %)			<0.001
Normal weight (<25.0 Kg/m^2^)	682 (42.8%)	40 (7.1%)	
Overweight (25.0–29.9 Kg/m^2^)	604 (35.5%)	149 (26.9%)	
Obese (≥30.0 Kg/m^2^)	399 (21.7%)	304 (66.1%)	
LDL-c, mg/dL (Mean ± SE)	117.33 ± 1.22	120.00 ± 1.81	0.219
Total cholesterol, mg/dL (Mean ± SE)	198.25 ± 1.35	207.75 ± 2.09	<0.001
CRP, mg/dL (Mean ± SE)	0.40 ± 0.03	0.56 ± 0.04	0.119

* Weighted column percentage. Weighted *p*-values were based on one-way ANOVA tests for continuous variables and Rao–Scott chi-square tests for categorical variables. BMI, body mass index; CRP = C-reactive protein; DBP, diastolic blood pressure; HDL-c, high-density lipoprotein cholesterol; HOMA-IR, homeostasis model assessment-estimated insulin resistance; LDL-c, low-density lipoprotein cholesterol; MET, metabolic equivalent task; MetS, metabolic syndrome; SBP = systolic blood pressure; SE = standard error. Different categories are indicated by bold subheadings.

**Table 2:** Coffee and caffeine intake by metabolic phenotype.

Variables	Metabolic Phenotype *		Weighted *p*-Value
	Metabolic Healthy Phenotype (MHP) (*n* = 1708)	Metabolic Unhealthy Phenotype (MUHP) (*n* = 493)	
**Coffee Consumption (*n*, %)**			0.513
None or ≤1 cup/week	730 (41.3%)	174 (37.0%)	
2–6 cups/week	199 (11.4%)	57 (10.6%)	
1 cup/day	274 (13.5%)	84 (14.3%)	
≥2 cups/day	505 (33.8%)	178 (38.0%)	
**Caffeine, mg/day (Mean ± SE)**	180.91 ± 6.47	193.38 ± 11.54	0.267
**Caffeine**			0.153
<35.3 mg/day	435 (22.3%)	112 (17.8%)	
35.5 mg/day–<106.5 mg/day	432 (22.1%)	111 (20.0%)	
106.5 mg/day–<219.5 mg/day	426 (25.7%)	136 (28.1%)	
≥219.5 mg/day	415 (29.8%)	134 (34.1%)	

* Weighted column percentage. Weighted *p*-values were based on one-way ANOVA tests for continuous variables and Rao–Scott chi-square tests for categorical variables. SE, standard error. Different categories are indicated by bold subheadings.

**Table 3:** Association of metabolic variables, number of metabolic abnormalities, and plasma folate and vitamin B12 levels with coffee consumption.

Metabolic Variables (*n*, %)	Coffee Consumption *				
	None/≤1 cup/week (904, 41.1%)	2–6 cups/week (256, 11.6%)	1 cup/day (358, 16.3%)	≥2 cups/day (683, 31%)	Weighted *p*-Value
BMI, Kg/m^2^ (Mean ± SE)	28.78 ± 0.32	28.45 ± 0.43	27.79 ± 0.47	27.62 ± 0.22	<0.001
SBP, mm Hg (Mean ± SE)	119.96 ± 0.93	121.39 ± 1.35	120.90 ± 1.21	122.83 ± 0.87	0.027
DBP, mm Hg (Mean ± SE)	70.64 ± 0.53	70.24 ± 0.70	69.43 ± 0.89	70.44 ± 0.40	0.568
Glucose, mg/dL (Mean ± SE)	94.43 ± 0.53	95.82 ± 0.73	95.37 ± 0.94	95.61 ± 0.44	0.020
HDL-c, mg/dL (Mean ± SE)	53.42 ± 0.99	55.47 ± 1.24	55.94 ± 1.39	55.63 ± 1.14	0.166
LDL-c, mg/dL (Mean ± SE)	114.31 ± 1.56	116.43 ± 1.84	120.06 ± 2.21	121.59 ± 1.62	<0.001
Total cholesterol, mg/dL (Mean ± SE)	195.31 ± 1.70	199.36 ± 2.68	202.05 ± 2.65	205.59 ± 1.77	<0.001
Triglyceride, mg/dL (Mean ± SE)	137.70 ± 4.46	135.24 ± 7.85	132.99 ± 5.82	133.79 ± 3.81	0.522
HOMA-IR (Mean ± SE)	16.53 ± 0.99	14.69 ± 0.75	13.96 ± 1.04	12.33 ± 0.41	<0.001
CRP, mg/dL (Mean ± SE)	0.49 ± 0.05	0.47 ± 0.05	0.42 ± 0.04	0.36 ± 0.04	0.021
**Number of metabolic abnormalities**					0.871
0	298 (34.4%)	81 (35.1%)	102 (35.6%)	175 (30.5%)	
1	259 (28.1%)	71 (28.0%)	100 (24.8%)	173 (25.3%)	
2	173 (18.2%)	47 (17.0%)	72 (17.7%)	157 (21.1%)	
3	112 (11.6%)	32 (11.9%)	49 (13.9%)	98 (13.4%)	
4	51 (6.3%)	21 (6.6%)	28 (6.8%)	64 (7.6%)	
5	11 (1.3%)	4 (1.3%)	7 (1.3%)	16 (2.1%)	
**Folate, ng/mL** **(Mean** **± SE)**	12.69 ± 0.39	12.38 ± 0.47	14.20 ± 0.68	14.00 ± 0.49	0.009
**Folate, ng/mL**					0.009
<8.3	244 (25.5%)	66 (25.0%)	82 (21.0%)	146 (22.9%)	
8.3–<11.5	244 (28.1%)	74 (29.8%)	81 (22.5%)	146 (21.8%)	
11.5–<16.1	206 (23.3%)	63 (27.5%)	105 (30.8%)	176 (25.9%)	
≥16.1	200 (23.0%)	49 (17.7%)	88 (25.8%)	211 (29.5%)	
**Vitamin B12, pg/mL** **(Mean ± SE)**	560.65 ± 35.69	487.32 ± 14.25	539.05 ± 19.39	509.55 ± 15.32	0.218
**Vitamin B12, pg/mL**					0.475
<359.0	203 (25.7%)	73 (29.1%)	83 (25.1%)	179 (28.3%)	
359.0–<470.5	218 (25.6%)	68 (29.9%)	77 (22.6%)	177 (26.0%)	
470.5–<634.5	227 (24.0%)	55 (22.3%)	96 (26.9%)	169 (24.2%)	
≥634.5	236 (24.7%)	55 (18.6%)	97 (25.5%)	147 (21.5%)	

* Weighted column percentage. Weighted *p*-values were based on one-way ANOVA tests for continuous variables and Rao–Scott chi-square tests for categorical variables. BMI, body mass index; CRP = C-reactive protein; DBP, diastolic blood pressure; HDL-c, high-density lipoprotein cholesterol; HOMA-IR, homeostasis model assessment-estimated insulin resistance; LDL-c, low-density lipoprotein cholesterol; MET, metabolic equivalent; MetS, metabolic syndrome; SBP = systolic blood pressure; SE = standard error. Different categories are indicated by bold subheadings.

**Table 4:** Associations between coffee consumption, caffeine intake, and metabolic phenotypes based on BMI status.

Variable	Total (*n* = 2201)	BMI Status *								
		Normal Weight (*n* = 722)			Overweight (*n* = 753)			Obese (*n* = 703)		
		MHP (*n* = 682)	MUHP (*n* = 40)	Weighted *p*-Value	MHP (*n* = 604)	MUHP (*n* = 149)	Weighted *p*-Value	MHP (*n* = 399)	MUHP (*n* = 304)	Weighted *p*-Value
**Coffee Consumption (*n*, %)**				0.98			0.015			0.56
None	584 (26.6%)	173 (95.4%)	10 (4.6%)		156 (91.6%)	18 (8.4%)		134 (57.7%)	88 (42.3%)	
		Ref			Ref			Ref		
≤1 time/week	320 (13.8%)	111 (95.7%)	6 (4.3%)		78 (86.2%)	10 (13.8%)		67 (59.7%)	42 (40.3%)	
		OR [95% CI] 0.92 [0.27, 3.17]			OR [95% CI] 1.75 [0.67, 4.56]			OR [95% CI] 0.92 [0.52, 1.63]		
2–6 times/week	256 (11.2%)	69 (97.2%)	4 (2.8%)		78 (87.2%)	16 (12.8%)		50 (57.8%)	37 (42.2%)	
		OR [95% CI] 0.60 [0.15, 2.44]			OR [95% CI] 1.60 [0.63, 4.08]			OR [95% CI] 0.99 [0.55, 1.81]		
1 time/day	358 (13.7%)	108 (95.4%)	7 (4.6%)		102 (79.7%)	31 (20.3%)		59 (51.1%)	46 (48.9%)	
		OR [95% CI] 1.00 [0.26, 3.77]			OR [95% CI] 2.77 [1.24, 6.20]			OR [95% CI] 1.30 [0.73, 2.34]		
≥2 times/day	683 (34.6%)	221 (95.7%)	13 (4.3%)		190 (77.2%)	74 (22.8%)		89 (49.9%)	91 (50.1%)	
		OR [95% CI] 0.91 [0.31, 2.72]			OR [95% CI] 3.22 [1.62, 6.39]			OR [95% CI] 1.37 [0.86, 2.19]		
Caffeine, mg/day (Mean ± SE)	183.53 ± 6.31	164.99 ± 9.40	147.67 ±40.46	0.69	204.79 ± 6.53	220.39 ± 16.40	0.37	168.85 ± 13.36	187.30 ± 16.30	0.40
		OR [95% CI] for one SD increase 0.90 [0.50, 1.61]			OR [95% CI] for one SD increase 1.06 [0.90, 1.25]			OR [95% CI] for one SD increase 1.11 [0.92, 1.34]		

* Weighted column percentage. Weighted *p*-values were based on one-way ANOVA tests for continuous variables and Rao–Scott chi-square tests for categorical variables. SE, standard error, Ref, reference group, OR, odds ratio, CI, confidence interval. Different categories are indicated by bold subheadings.

**Table 5:** Correlations between plasma folate and vitamin B12 levels with metabolic phenotypes.

Variables	Metabolic Phenotype *		Weighted *p*-Value
	Metabolic Healthy Phenotype (MHP) (*n* = 1708)	Metabolic Unhealthy Phenotype (MUHP) (*n* = 493)	
**Folate, ng/mL (Mean ± SE)**	12.97 ± 0.32	14.65 ± 0.69	0.004
**Folate**			0.277
<8.3 ng/mL	433 (24.3%)	105 (22.3%)	
8.3 ng/ML–<11.5 ng/mL	433 (25.7%)	112 (23.9%)	
11.5 ng/mL–<16.1 ng/mL	422 (25.9%)	128 (25.0%)	
≥16.1 ng/mL	406 (24.0%)	142 (28.8%)	
**Vitamin B12, pg/mL (Mean ± SE)**	541.64 ± 20.53	494.41 ± 13.40	0.035
**Vitamin B12**			0.027
<359.0 pg/mL	401 (25.8%)	137 (31.2%)	
359.0 pg/Ml–<470.5 pg/mL	415 (25.1%)	125 (28.5%)	
470.5 pg/mL–<634.5 pg/mL	431 (25.1%)	116 (21.1%)	
≥634.5 pg/mL	430 (24.0%)	105 (19.3%)	

* Weighted column percentage. Weighted *p*-values were based on one-way ANOVA tests for continuous variables and Rao–Scott chi-square tests for categorical variables. SE, standard error. Different categories are indicated by bold subheadings.

**Table 6:** Associations between plasma folate and vitamin B12 and metabolic phenotypes, based on BMI status.

Variable	Total (*n* = 2201)	BMI Status *								
		Normal Weight (*n* = 722)			Overweight (*n* = 753)			Obese (*n* = 703)		
		MHP (*n* = 682)	MUHP (*n* = 40)	Weighted *p*-Value	MHP (*n* = 604)	MUHP (*n* = 149)	Weighted *p*-Value	MHP (*n* = 399)	MUHP (*n* = 304)	Weighted *p*-Value
**Folate, ng/mL** **(Mean ± SE)**	13.32 ± 0.34	13.43 ± 0.34	16.55 ± 1.81	0.11	13.44 ± 0.38	15.97 ± 1.19	0.020	11.30 ± 0.49	13.91 ± 0.67	0.0006
		OR [95% CI] for one SD increase 1.27 [0.94, 1.70]			OR [95% CI] for one SD increase 0.27 [0.10, 2.61]			OR [95% CI] for one SD increase 1.47 [1.17, 1.84]		
**Vitamin B12, pg/mL** **(Mean ± SE)**	531.71 ± 17.55	548.70 ± 12.96	550.92 ± 48.40	0.97	571.49 ± 53.36	506.16 ± 24.85	0.25	482.22 ± 17.43	483.26 ± 18.62	0.96
		OR [95% CI] for one SD increase 1.04 [0.16, 6.64]			OR [95% CI] for one SD increase 0.67 [0.19, 2.38]			OR [95% CI] for one SD increase 1.02 [0.46, 2.25]		

* Weighted column percentage. Weighted *p*-values were based on one-way ANOVA tests for continuous variables and Rao–Scott chi-square tests for categorical variables. SE, standard error, OR, odds ratio, CI, confidence interval. Different categories are indicated by bold subheadings.

## Data Availability

The NHANES 2003–2006 data used in the current manuscript can be downloaded from CDC website: https://wwwn.cdc.gov/nchs/nhanes/Default.aspx, accessed on 16 April 2021.
